# Emerging Functions for Cell Wall Polysaccharides Accumulated during Eudicot Seed Development

**DOI:** 10.3390/plants7040081

**Published:** 2018-09-29

**Authors:** Julien Sechet, Annie Marion-Poll, Helen M. North

**Affiliations:** Institut Jean-Pierre Bourgin, INRA, AgroParisTech, CNRS, Université Paris-Saclay, 78000 Versailles, France; Julien.Sechet@inra.fr (J.S.); Annie.Marion-Poll@inra.fr (A.M.-P.)

**Keywords:** cell wall, pectin, cellulose, embryo, endosperm, hemicelluloses, polysaccharides, seed, seed coat

## Abstract

The formation of seeds is a reproductive strategy in higher plants that enables the dispersal of offspring through time and space. Eudicot seeds comprise three main components, the embryo, the endosperm and the seed coat, where the coordinated development of each is important for the correct formation of the mature seed. In addition, the seed coat protects the quiescent progeny and can provide transport mechanisms. A key underlying process in the production of seed tissues is the formation of an extracellular matrix termed the cell wall, which is well known for its essential function in cytokinesis, directional growth and morphogenesis. The cell wall is composed of a macromolecular network of polymers where the major component is polysaccharides. The attributes of polysaccharides differ with their composition and charge, which enables dynamic remodeling of the mechanical and physical properties of the matrix by adjusting their production, modification or turnover. Accordingly, the importance of specific polysaccharides or modifications is increasingly being associated with specialized functions within seed tissues, often through the spatio-temporal accumulation or remodeling of particular polymers. Here, we review the evolution and accumulation of polysaccharides during eudicot seed development, what is known of their impact on wall architecture and the diverse roles associated with these in different seed tissues.

## 1. Introduction

Plant cells are distinguished by a combination of features, including chloroplasts, large central vacuoles and cell walls. The latter are formed of an extracellular matrix of polymers that can adhere cells to one another; cell walls also provide mechanical resistance and define shape thereby influencing overall plant structure. Consequently, for cell expansion and division the properties and interactions between polymers must be modulated so that the wall can be more elastic. When this occurs in a localized manner it dictates the direction of growth, thus the remodeling of cell walls plays a fundamental role in the control of growth and development [1]. 

A key step in higher plant development is reproduction through seeds, and the formation of this new organ requires massive production of cell walls for the generation of the embryo, endosperm and protective maternal tissues. As reproduction is a critical phase in a plant’s lifecycle, understanding the mechanisms underlying cell wall production and remodeling during seed development is a major challenge. Moreover, modified seed development can have knock-on effects on germination speed and synchronicity, which consequently impact yield and plant fitness [2]. For this reason, genes affecting seed development through cell wall production are potential targets for crop improvement. 

The principal constituents of cell walls are the polysaccharides cellulose, hemicellulose and pectin. Cellulose microfibrils have a relatively simple primary structure, being formed of 18–24 unbranched (1→4)-β-D-glucan chains that are aligned during their synthesis and can aggregate to form partially crystalline structures through inter-molecular hydrogen bonds and hydrophobic interactions [3]. Renowned for their mechanical resistance, cellulose microfibrils have hydrophobic and hydrophilic surfaces whose availability for interaction with other polymers is influenced by chain packing. Hemicelluloses are more diverse with xyloglucan (XyG) being predominant in eudicot primary cell walls [4]. XyG has a cellulose-like backbone of (1→4)-β-D-glucan that is substituted with a variety of xylose containing side groups that are distributed along the backbone with a degree of regularity. The xylosyl side groups can be substituted further with β-D-galactosyl, and this can sometimes have an additional α-L-fucosyl substitution [5,6]. The presence of the side chains inhibits xyloglucan aggregation and interactions with other polysaccharides, while regions with no substitution have a high affinity for cellulose and could act as a mechanical linker between adjacent cellulose microfibrils [3,7]. Similarly, the precise patterning of glucomannan substitutions with α-D-galactosyl could stabilize the interaction of this hemicellulose with cellulose, thereby modulating microfibril aggregation [8]. Pectin is a copolymer with varied domains that all contain galacturonic acid (GalA), the simplest being homogalacturonan (HG) which can be substituted with rhamnose (Rha) containing branches to form rhamnogalacturonan II (RGII) domains [9]; the latter are, however, a relatively minor component of cell walls. In contrast, rhamnogalacturonan I (RGI) domains have a backbone formed of a repeating disaccharide comprising →2) 2-α-L-Rha-(1→4)-α-D-GalA-(1→ which can be decorated with arabinan, galactan, arabinogalactan or occasionally xylan [10,11]. Other polysaccharides are also observed in certain seed cell walls, for example callose, a (1→3)-β-D-glucan whose properties contribute to reducing wall permeability [12]. Our current knowledge of the polysaccharide biosynthesis machinery has been the subject of a recent review [13] and will not be reiterated here.

The different polymers present in the cell wall form a network whose functional features are not only determined by individual polysaccharide properties, but also their capacity to link and aggregate. For example, the alignment of cellulose microfibrils can create mechanical anisotropy and direct growth, while the formation of hemicellulose crosslinks between cellulose microfibrils contributes to wall reinforcement and spacing [1]. Crosslinks can also be formed within the pectin matrix between demethylesterified HG blocks through Ca^2+^ bonds, or RGII molecules with borate-ester bonds. There is also evidence that RGI adsorbs to cellulose through arabinan, galactan, or xylan side chains [11,14]. It is well documented that the composition of primary and secondary cell walls differ, the latter having less pectin and more hemicellulose and containing other polymers such as lignin [15]. The type and amount of each polysaccharide is also heterogeneous between species and tissues. This influences the interactions and bonds that are formed within a given matrix between polymers and the resulting mechanical properties. The current review spotlights how accumulation, interactions and remodeling of cell wall polysaccharides influence seed development and physiology, focusing on knowledge obtained using Arabidopsis, with selected examples from crop eudicots.

## 2. General Overview of Polysaccharide Production during Arabidopsis Seed Development 

**Figure 1:**
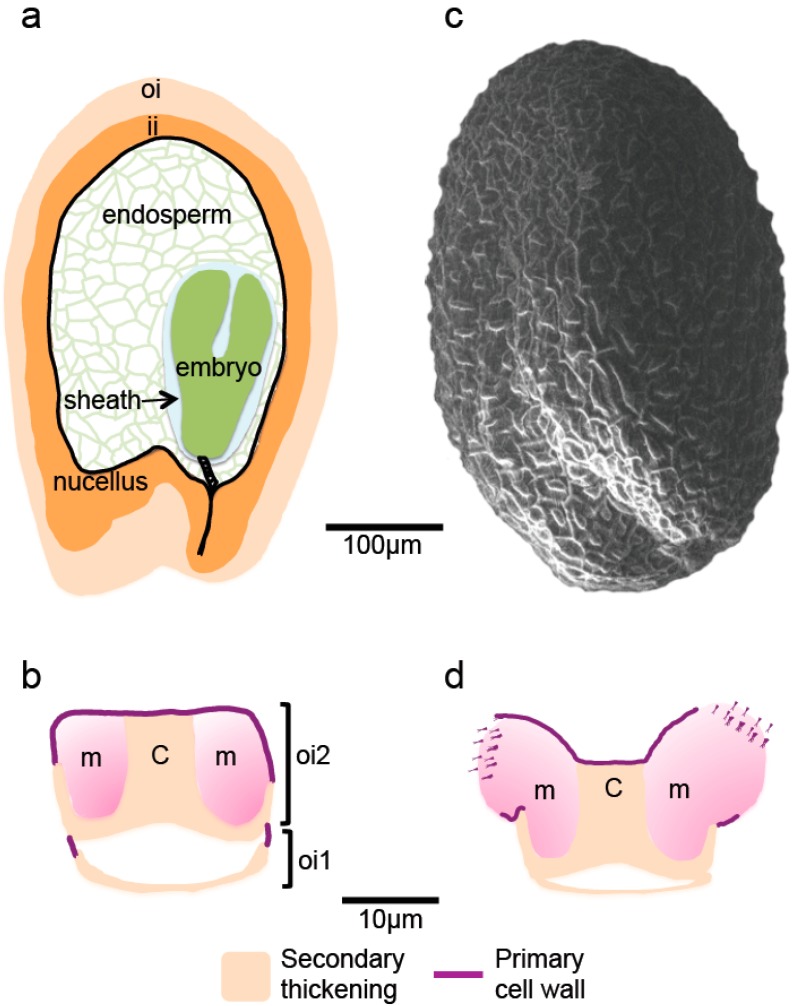
Structure of Arabidopsis seed and outer integument cells. Schematic representation of (**a**) the different tissues in a transverse section of a developing seed at the linear cotyledon stage when the endosperm has cellularized and (**b**) transverse section of fully differentiated outer integument cells from early maturation stage seeds. The cells have undergone programmed cell death, following the accumulation of mucilage polysaccharides and/or secondary thickening of the innermost walls. (**c**) Scanning electron micrograph of the reticulate surface of a mature dry seed. (**d**) Schematic representation of the localized fragmentation of the primary wall on imbibition that allows mucilage release from the epidermal cells of the seed coat. C, columella, m, mucilage polysaccharides, oi, outer integuments, ii inner integuments and oi1 and oi2, sub-epidermal and epidermal cells of the outer integument, respectively.

Like other eudicots, the Arabidopsis seed is comprised of an embryo that gives rise to the future plant, the endosperm, a nutritive tissue, and a protective seed coat (Figure 1a). Parallel fertilization events give rise to the triploid endosperm, whose genome contains two copies of the maternal genome and one of the paternal genome, and the diploid embryo. First embryo divisions form from the basal cell the suspensor and the hypophysis, and from the apical cell the embryo proper. Subsequent embryogenesis can be broadly divided into 5 morphogenetically distinct stages: globular, heart, linear-cotyledon, bent-cotyledon and maturation. The division and patterning processes involved have been well defined in Arabidopsis and details can be found in an excellent review by ten Hove et al. [16]. Analogous to somatic cells, embryo cell wall formation takes place during cell division. A cortical ring of cytoskeletal filaments called the preprophase band (PPB) is formed transiently and marks the position of the future division plane and new cell walls. During mitosis, the phragmoplast and cell plate are formed and expand until the cell plate fuses to the existing cell walls [17]. The formation of this cell plate involves the transport and deposition of polysaccharides by Golgi-derived vesicles and it has recently been established that cellulose synthase complexes are also transported and active from early stages of cell plate formation [18]. In contrast, endosperm nuclei undergo mitotic divisions without cell wall biogenesis, leading to the formation of a large cell containing multiple nuclei mostly located at the periphery of the endosperm and surrounding a central vacuole. After a series of eight syncytial divisions cellularization of the endosperm begins; at this time, the embryo is at the globular stage [19,20]. This *de novo* cell wall formation takes place in the absence of a PPB and involves the formation of phragmoplast-like structures and syncytial-type cell plates [21,22]. After cellularization, the cell division rate decreases and the endosperm is completely or partially absorbed by the growing embryo. In Arabidopsis, the endosperm mainly persists as a single peripheral layer. It has been suggested that cell wall modifications that occur prior to endosperm reduction might be an upstream factor of cell elimination [23], as discussed further below. 

The seed coat develops in parallel to embryo morphogenesis and as this is derived from differentiation of the cells of the ovule it is a maternal tissue. Two components can be distinguished, the integuments and the nucellus (Figure 1a), which corresponds to the residual megasporangium. While the latter is reduced by nearly 50% over the first few days following fertilization by programmed cell death (PCD) [24], the integuments undergo significant growth. Considerable synthesis of primary cell walls occurs in the teguments over this period with the mature size being reached within the first few days of seed development, well in advance of embryo growth [25]. This is driven by cell expansion and division in the three inner and two outer tegument layers accompanied by an increase in the volume of the central vacuoles. In contrast to the inner layers, the two cell layers of the outer integument then accumulate starch granules followed by the accumulation of polysaccharides. For the innermost layer, this results in the formation of a thickened cell wall juxtaposing the inner integument (Figure 1b), while the epidermal cell layer accumulates polysaccharides in the apoplast that will be released on imbibition to form a hydrogel termed mucilage. This layer is subsequently reinforced by the progressive deposition of secondary cell wall material replacing the cytoplasm that has been forced into a column and forming a continuum along the inner face of the cell and part way up the radial cell walls, which generates the distinctive reticulated surface of Arabidopsis seeds (Figure 1b,c). On completion of seed coat differentiation, shortly after reserve accumulation has begun during seed maturation, seed coat cells have either been crushed or undergone PCD, so no further remodeling of polysaccharides is possible and properties are fixed. It should be noted that natural variation is observed in the number of seed coat cell layers, with the reference accession Columbia generally being formed of five cell layers, while the Wassilewkija accession more frequently has six [26]. 

## 3. Localized Accumulation and Remodeling of Cell Wall Polysaccharides during Eudicot Seed Development 

### 3.1. Distinctive Polysaccharide Accumulation Patterns in the Embryo and Endosperm

Variations in the temporal and spatial distribution of polysaccharides during seed development have been reported by a number of studies, suggesting significant cell wall remodeling occurs and a role for cell wall modifications in seed physiology (Table 1). In the embryo, cell wall biogenesis during cytokinesis is similar to that observed in somatic cells. Transient callose synthesis and deposition have been observed within cell plates and suggested to contribute to fluidity and enlargement of the membrane network in immature cell plates. The removal of callose and deposition of cellulose and pectin would then stiffen the mature cell plates [27,28]. In the endosperm callose deposition has also been reported in syncytial-type cell plates; however, its persistence in mature Arabidopsis endosperm cell walls while cellulose levels remain low, has been suggested to have additional beneficial roles. For example, it might constitute a more readily available carbohydrate reserve compared to cellulose and/or confer plasticity to endosperm cell walls during embryo growth [29]. Immunolabeling studies uncovered other specificities of cell walls in the developing endosperm of Arabidopsis at early stages: a lower cellulose content and the complete absence of fucosylated XyG compared to cell walls of other seed tissues. Similar to callose, it has been suggested that fucose-less XyG may have a specific function in the endosperm in relation to embryo growth, but no developmental defect has yet been reported in seeds with altered XyG fucosylation [21,30]. The reduction of XyG side chain length has been proposed to require less extensive enzymatic machinery thereby enabling rapid turnover [4]. At later stages of seed development, cellulose and all types of substituted XyG were observed in every seed tissue including endosperm. Moreover, epitope-labeling detected higher amounts of fucosylated XyG in endosperm cell walls compared to those of the embryo [31]. In mature dry seeds, embryo cell walls were reported to contain more XyG and cellulose than those of the endosperm, but lower amounts of de-esterified HG and arabinans [32]. Other recent studies suggested the involvement of the degree of pectin methyl esterification in cell wall remodeling during endosperm development. Whereas at torpedo stage, both high- and low- methylesterified HG were barely detectable, in bent cotyledon stage embryos, low- and de-esterified HG epitopes were strongly labeled in the endosperm. A low degree of pectin methylesterification has been hypothesized to contribute to endosperm weakening, which as a result facilitates embryo expansion [33]. Together these spatiotemporal variations in relative abundance and structure of cell wall polysaccharides suggest a distinct developmental control of cell wall characteristics in embryo and endosperm from fertilization to seed desiccation.

Endosperm architecture and the spatial distribution of its cell wall polysaccharides in mature seeds have also been reported to vary between species and influence germination. In Arabidopsis and cress (*Lepidium sativum*), a related Brassicaceae species, micropylar endosperm is formed of more cell layers than the rest of the endosperm and this is likely to contribute to mechanical resistance against radicle protrusion, whereas the tobacco endosperm is uniformly composed of three to five layers [38]. Nevertheless, in contrast to Arabidopsis and cress seeds, asymmetry was found in the cell wall composition of the tobacco micropylar endosperm, which exhibited an increased abundance of XyG and RGI epitopes. A subsequent study reported the predominance of arabinan-rich RGI, which might affect local cell wall mechanical properties and condition radicle emergence [32]. Another special feature of the tobacco endosperm, compared to Arabidopsis and cress, was the presence of heteromannans. While these hemicelluloses are evenly distributed in the mature endosperm, they are specifically degraded in the micropylar zone during germination [38]. Similar to tomato endosperm, mannan abundance contributes to the rigidity of endosperm tissues, which upon seed hydration are also hydrolyzed in the micropylar region by the spatially restricted activation of cell wall remodeling enzymes [47].

Specific pectin accumulation patterns have been shown to influence cell wall rigidity/flexibility not only in the endosperm, but also in the embryo. Indeed, the deposition of (1→4)-β-galactans has been observed in pea cotyledon cell walls at a defined stage late in development, whereas HG and (1→5)-α-arabinans were detected throughout seed development. The presence of (1→4)-β-galactans was restricted to a distinct thin layer at the inner face of the pea cotyledon cell walls and was linked to increased mechanical resistance to compression [56], whereas in lupin (1→4)-β-galactans were found to be massively accumulated in cotyledon cell walls as a storage polysaccharide. As extensively reviewed by Buckeridge [57], in a number of plant species embryo or endosperm cell wall polysaccharides can also act as the principal seed reserve. XyG and arabinogalactans are mainly used as reserves in cotyledons, whereas mannans, glucomannans and galactomannans are stored in the endosperm. These hemicelluloses are then mobilized during germination. Interestingly, reserve XyG differs from that in primary walls as it is not substituted with terminal α-L-fucosyl [57]. Seeds of very diverse species accumulate cell wall polysaccharides as a storage reserve, indicating that this trait arose many times independently in plant evolution [58]. 

### 3.2. Specialization of the Seed Coat through Polysaccharide Accumulation

In the seed coat, while mucilage composition has been well described for a number of species only limited information is available concerning the polysaccharide components of reinforced walls in seed coats. Published images of immunolabeled sections of mature Arabidopsis seeds [31] indicate that the XyG contents of the outer primary wall of the epidermal cells is much higher than the other residual walls of the seed coat, implying that the latter are mainly secondary wall polysaccharides. In addition, histochemical stains have indicated that the secondary thickening of columella and outer integument cell walls is rich in cellulose, with callose also observed in the former [49]. Localized callose accumulation has also been reported in reinforced cell walls of sclereids in the soybean seed coat, with the walls orientated towards the outside of the seed being brightly labeled by the callose-specific stain aniline blue [59]. 

Seed mucilage can vary greatly in both composition and structure between and within species with a major distinction being whether it is cellulosic [60]. Non-cellulosic mucilage can be easily extracted by rapid agitation in water and in psyllium (*Plantago ovata*) is composed mainly of heteroxylan [55]. In contrast, the major component of Arabidopsis mucilage is RGI, and while two-thirds is essentially unsubstituted and can be removed from the seed by water-extraction, the remainder is tightly attached to the seed coat through xylan substitutions that adsorb to the comparatively small amounts of cellulose [11,49]. An intermediate composition is observed for commercial flaxseed (*Linum usitatissimum*) mucilage, which is rich in both arabinoxylan and RGI [39]. In addition to cellulose the presence of other minor polysaccharides can also have significant effects on mucilage structure, for example in Arabidopsis the amount of galactoglucomannan and RGI branched with arabinan or galactan influence the density of the mucilage gel [40,41,42,48,61]. Seed coat epidermal cells can also accumulate very large quantities of cellulose, notably, as cotton fibers derived from the thickened walls of elongated unbranched trichomes. Due to the commercial importance of cotton, the accumulation and modeling of polysaccharides has been studied in detail in these cells and shows some interesting particularities. Notably, polysaccharide remodeling through pectin and XyG degradation in the adhesive middle lamella allows adjacent fiber cells to be individualized and the induction of large scale cellulose synthesis during the transition to secondary wall thickening [54]. 

Localized remodeling of the primary cell wall has also been found to be important for mucilage release from the epidermal cells of Arabidopsis seed coats. When mucilage polysaccharides are hydrated they expand rapidly and their release is facilitated by the rupture of the outer primary cell wall at its junction with the radial cell wall (Figure 1d). During seed development, limiting HG demethylesterification and localized oxidation of primary cell wall components in this zone appear to be imperative for fragmentation to occur on subsequent imbibition [36,62,63]. 

The accumulation of certain polysaccharides in either thickened cell walls or mucilage at specific developmental stages involves the massive induction of corresponding biosynthesis pathways. As the developmental point of this induction can be easily tracked from the time of fertilization, a number of species have served as models for the identification of novel biosynthesis genes using transcriptome analyses [64,65,66,67]. Furthermore, in laboratory conditions the absence of mucilage accumulation and cell wall thickening in seed coats is not detrimental to subsequent embryo survival and has allowed numerous mutants to be identified which have been used for the identification of genes involved in polysaccharide production as well as functional analyses [68]. 

## 4. Functional Roles of Polysaccharides during Seed Development

### 4.1. Cell Wall Biosynthesis Defects that Induce Embryo Lethality

The essential role of the cell wall in the determination of shape and growth can hinder functional analyses of the contribution of polysaccharides to seed development and physiology. In effect, embryo lethality has been reported for a number of mutants affected in the production of polysaccharides, and while this implies that the corresponding genes play crucial roles in cell division and elongation during embryo growth, characterization of the precise function is limited (Table 1). For example, cellulose is the main structural component of the cell wall and null mutant alleles of *CELLULOSE SYNTHASE1* (*CESA1*) and *CESA3* are gametophytic lethal due to the essential role of these subunits in primary wall synthesis [69]. Whilst the missense mutant *cesa1^rsw^*^1*-*2^ is embryo-lethal, the radial swelling of the embryo demonstrated the role of differential wall reinforcement by cellulose in the orientation of anisotropic cell elongation during morphogenesis and organ expansion [50]. Similar phenotypes were also observed in a second mutant with defective cellulose synthesis, *knf1*. Furthermore, a number of CELLULOSE SYNTHASE-LIKE A (CSLA) proteins have been shown to have mannan synthase activity [70,71] and carry out glucomannan synthesis *in planta* [44,45]. While mutation of *CSLA7* triggered embryo lethality with a developmental arrest at the globular stage [46], over-expression of *CSLA2*, *CSLA7* and *CSLA9* also arrested or delayed embryo development suggesting that the fine-tuning of mannan contents is important during embryogenesis [45]. Mannan would also appear to contribute to the formation of endosperm cell walls, as cellularization did not take place in *csla7* mutant seeds [46]. 

### 4.2. Polysaccharides in Seed Cell Division, Expansion and Elimination

A few mutants have been identified that affect polysaccharide properties in embryo and endosperm without causing lethality and these are proving to be precious tools for obtaining detailed information about roles of the cell wall in seed development and physiology (Table 1). For example, disrupting the formation of new cell walls at the cell plate during cytokinesis is bound to cause major defects and while null mutants for the callose synthase GSL8 (*massue*/*gsl8*) have altered cell division planes and occasionally multi-nucleated cells due to delayed callose deposition at the cell plate, the resulting embryos germinate despite being malformed [28,72]. This mutant confirmed the role of callose described above as a transient actor in cell division. Similarly, mutation of the gene encoding the cell wall protein EXTENSIN3/ROOT-SHOOT-HYPOCOTYL DEFECTIVE also induces defective positioning of the cell plate in the *rsh* mutant from as early as the first asymmetric division of the zygote [43]. It seems likely, therefore, that the formation of the nascent cell plate involves interactions between extensins and pectin [44]. In *mas/gsl8* and *rsh* mutants, development is nonetheless sufficiently affected that it is seedling-lethal.

Pectin composition and abundance have been associated with modifications to mechanical properties of embryo and endosperm cell walls, as mentioned above, and this has been confirmed through functional genetic approaches. When the pectin methylesterase inhibitor PMEI5 was over-expressed to maintain a higher level of HG methylation in plant tissues, transformants were found to have bigger seeds with larger cells in the embryo, endosperm and seed coat [34]. Conversely, when a high degree of HG methylesterification was obtained in embryo tissues through mutation of the pectin methylesterase HIGHLY METHYL ESTERIFIED SEEDS (HMS) this resulted in delayed embryo development and morphogenesis, and seeds and cell size were smaller [35]. While these studies demonstrate that the degree of pectin methylesterification is a determinant of cell expansion during seed development, they indicate contradictory effects for high HG methylesterification. This would appear to be due to the existence of several pathways that remodel seed pectin since overexpression of PMEI5 in *hms* rescued the developmental defects, indicating that HMS and PMEI5 targets act independently in HG remodeling [35] and that their action affects HG properties differently. The DUF642 cell wall-associated protein, BIIDXI, is a further regulator of pectin methylesterase activity required for normal embryo development, as embryo bending is impaired in mutant seeds [33]. Here, however, differences in methylesterification appeared to be correlated with less demethylesterifed HG in endosperm cell walls. This suggested that in addition to controlling embryo cell expansion, pectin remodeling during seed development can also impact the elasticity of the endosperm and its ability to be degraded to make room for the growing embryo. Indeed, cell wall rheology is dependent on the dimerization status of demethylesterified HG through Ca^2+^ crosslinks. Altered endosperm breakdown has also been correlated with modified cell wall composition and properties in the *zhoupi (zou)* mutant, notably the maintenance of arabinan in walls adjacent to the embryo which is likely to be present as RGI branches [37]. ZOU is a basic helix-loop-helix (bHLH) transcription factor that is specifically expressed in the endosperm of developing seeds [73] and which forms a heterodimer with a second bHLH, INDUCER OF CBP EXPRESSION 1 (ICE1), to trigger endosperm elimination [73,74]. Its role in the remodeling of pectin to facilitate endosperm cell compression by the growing embryo is, therefore, not as a direct actor on the cell wall, but the regulation of expression of cell wall remodeling enzymes or regulators. In addition, *zou* mutants exhibited adhesion between the surfaces of the embryo and surrounding endosperm cells, which were initially attributed to its requirement for the correct biogenesis of the embryo cuticle [75]. Immunolabeling experiments showed, however, that *zou* embryos were also defective for the production of an extracuticular sheath derived from the endosperm that contains epitopes recognized by the anti-extensin antibody JIM12 [76]. ZOU was shown to regulate the expression of KERBEROS, a peptide secreted by the endosperm, which is also required for the production of the embryo sheath, but is not the epitope recognized by JIM12 [76]. The exact composition of this sheath remains to be determined, but it could be glycoprotein-rich mucilage and represent a further function for polysaccharides during seed development, preventing abnormal adhesion of the embryo as it grows through the degenerating endosperm. 

Unexpectedly, seed developmental defects have not been described for any of the mutants identified to date affected in XyG biosynthesis or remodeling, despite this being the main hemicellulose of the primary cell wall. Nevertheless, germination defects have been observed that link XyG accumulation or side-chain trimming with the resistance of endosperm cell walls to radicle protrusion [31,38].

### 4.3. Diverse Functions for Seed Coat Polysaccharides

Mutants affected in the synthesis or remodeling of polysaccharides in the seed coat produce viable seedlings, thus indicating that the properties they confer to the seed benefit fitness rather than fulfilling essential functions. The diverse phenotypes observed for mutants affected in polysaccharide production have implicated them in a range of seed coat functions (Table 1). As the seed coat encases the embryo and endosperm it is evident that it plays a crucial role in their protection, limiting both physical damage and pathogen or predator accessibility. This protective function invokes a degree of mechanical resistance established through the reinforcement of cell walls by secondary thickening. It should be noted that the increased tensile strength of secondary cell walls is not simply a result of polysaccharide accumulation, but also the modification of composition and crosslinking to other polymers, such as lignin. Furthermore, in certain eudicots, additional maternal tissue from the ovaries is retained as the pericarp, which can also be reinforced with polysaccharides to increase the level of protection. In Arabidopsis, the importance of cellulose synthesis for secondary wall thickening of seed coat epidermal cells was confirmed by the observation of reduced radial wall reinforcement in single and mutant combinations affected in cellulose synthase catalytic subunits *CESA9*, *CESA2* and *CESA5* [51,52]. Similar defects have also been observed for *cinv1 cinv2*, defective for cytosolic invertases that generate UDP-glucose substrates for cellulose synthesis [53]. The precise function of the radial wall reticulations observed on Arabidopsis seeds remains to be determined, together with that of the intriguing central columella. 

Thickened seed coats can also be a barrier to germination, termed physical dormancy; either through physical resistance against radicle protrusion, or by impeding water transfer or gas exchange [77]. This can be alleviated by scarification or enzyme hydrolysis in the digestive system of an animal. The observation of reduced permeability in *cesa9* mutant seeds [51] is in agreement with a role for thickened seed coat cell walls in limiting interactions between the embryo/endosperm and the environment. This could be a factor in prolonging seed lifespan as hard coated seeds are often long lived [78]. A different function has been proposed for the reinforcement of the inner facing wall of the outer integument inner layer, which occurs earlier than that of the epidermal layer. Thickening in the former appears to be induced non-autonomously as a mechanosensitive response to endosperm and embryo growth, thereby blocking further seed enlargement and contributing to the control of seed size [79].

Seed dispersal in wild species is an important factor for the establishment of the future seedling as transport to new niches can improve fitness by limiting sibling density and competition [80]. Seed coat structures formed of polysaccharides have been proposed to aid dispersal; for example the fluffy hairs on cottonseed may aid wind dispersal [81]. Furthermore, defective mucilage release in natural accessions of Arabidopsis appears to be a local adaptation that maintains seed buoyancy and could impact water dispersal through flotation [82]. Mucilage polysaccharides can also act as bio-adhesives to bind soil particles in stable aggregates that consequently improve water and air flow properties [83], which is likely to benefit subsequent plant establishment. The stabilizing ability is likely to differ with variations in mucilage composition between species as soil-binding properties varies between polysaccharides [84].

## 5. Conclusions and Perspectives

The availability of mutants for the genetic manipulation of seed polysaccharides has enabled, recent studies to show that, in addition to an essential role in controlling cell growth and morphogenesis, polysaccharides have a range of additional functions during seed development. Furthermore, antibodies that recognize specific polysaccharide epitopes and histochemical stains have highlighted specific temporal and spatial accumulation of polysaccharides within seed tissues. This is likely to reflect the different properties endowed by each type of polymer, such as rigidity, flexibility, permeability and resistance to degradation. In addition to the enzymes that synthesize and trim polysaccharides, cell wall remodeling may also be modulated by trafficking mechanisms. Cell wall polymers can be secreted and recycled by the endomembrane system and this is a much neglected aspect of wall dynamics (reviewed in [85]) and during endosperm cell elimination, remobilization of cell wall components is likely to occur. The distinct architectures observed in different eudicot endosperm cell walls is particularly intriguing, and while genetic modification of Arabidopsis endosperm XyG contents has been found to modify germination rates [31,38] detailed analysis of the repercussions of changing cell wall composition during development remain to be determined. 

The role of the cell wall in communication between seed tissues leads to further questions concerning what are the underlying mechanisms that enable the chemical and physical status of walls to be signaled between cells. It is likely that during seed development equivalent cell wall integrity signaling mechanisms operate as those that function during cell expansion in other tissues. Cell wall integrity sensors have been identified as members of the receptor-like kinase (RLK) family where a unique class, termed wall-associated kinases (WAKs), has been shown to detect modifications in the pectin network (reviewed in [86]). RLKs also act as mechano- and osmo-sensors and could be important in the management of the mechanical confrontation of cell walls during embryo growth through the endosperm. 

The limited amount, or the limited accessibility of seed tissue has often hampered analyses of extracellular matrix composition or properties. The resolution of micro-imaging techniques, using for example ^1^H-NMR, Raman or FT-IR, is constantly improving, which is expected to make in situ detection of complex molecules feasible even in relatively small seeds [87,88]. Furthermore, new tools for analyzing the mechanical properties of such tissues are also being developed, such as Nano indentation [70]. The targeted production of multiple allelic-series of mutants through new-breeding technologies would also allow fine-tuning of mutational effects in genes that have embryo-lethal effects. This technique can also be used to stack mutations in multigene families where functional redundancy may mask phenotypes. Through the combination of these and other approaches we expect many of the secrets concerning polysaccharide functions in seed tissues to be revealed in the coming years.

## Figures and Tables

**Table 1:** Summary of cited data concerning the localization and function of cell wall polysaccharides during seed development.

Polysaccharide		Species	Tissue	Seed Developmental Stage when Detected	Known Function [Reference]
Pectin	HG	Arabidopsis	Embryo	Bent cotyledon, mature seed	Control of cell expansion by the degree of pectin methylesterification [34,35]
	Low- and de-esterified HG	Arabidopsis	Endosperm	Bent cotyledon, mature seed	Modulation of the elasticity of the endosperm and its ability to be degraded [33,35]
	Limited HG demethylesterification	Arabidopsis	Seed coat epidermal cells	Throughout development	Localized fragmentation of primary cell wall [36]
	Arabinan-rich RGI	Arabidopsis	Endosperm	Throughout development	Controlling endosperm breakdown [37]
	Arabinan-rich RGI	Arabidopsis Cress Tobacco	Endosperm	Mature seed	Mechanical resistance against radicle protrusion [32,38]
	RGI	Arabidopsis Flaxseed	Seed coat epidermal cells	From linear cotyledon to mature seed	Mucilage, reduction of seed buoyancy [39,40,41,42]
	Extensin/pectin interaction	Arabidopsis			Cytokinesis, cell plate formation [43,44]
Mannan		Arabidopsis	Embryo		Involved in embryogenesis [45,46]
		Arabidopsis Tobacco Tomato	Endosperm	Mature seed	Formation of endosperm cell walls (embryo lethal mutants) [46]. Rigidity of endosperm tissues, degraded to allow radicle protrusion [38,47]
	GGM	Arabidopsis	Seed coat epidermal cells	Mature seed	Mucilage structure [48]
Callose		Arabidopsis	Embryo	Transiently present in dividing cells	Cytokinesis, cell plate formation [28]
		Arabidopsis	Endosperm	Throughout development	Cytokinesis, cell plate formation [21,22]
		Arabidopsis	Seed coat epidermal cells	Mature seed	Columella formation [49]
		Soybean	Sclereids		
Xyloglucan	Galactosylated	Arabidopsis	Endosperm	Before endosperm cellularization	
	Galactosylated/Fucosylated	Arabidopsis	Embryo, seed coat	Before endosperm cellularization	
	Galactosylated/Fucosylated	Arabidopsis	Embryo Endosperm Seed coat epidermal cells	From bent cotyledon to mature seed	Mechanical resistance of endosperm against radicle protrusion [31]
Cellulose		Arabidopsis	Embryo	All seed stages	Orientation of anisotropic cell elongation and organ expansion [50]
		Arabidopsis	Endosperm	Low throughout development	
		Arabidopsis	Seed coat (outer integument and columella)	Throughout development	Mechanical resistance and columella formation [51,52,53]
		Arabidopsis	Seed coat epidermal cells	Mature seed	Attachment of mucilage pectin to seed coat [11,14]
		Cotton	Seed trichome	Throughout development	Cotton fiber [54]
Xylan		Arabidopsis	Seed coat epidermal cells	Mature seed	Attachment of mucilage pectin to seed coat [11]
	Heteroxylan	Psyllium	Seed coat epidermal cells	Mature seed	Mucilage [55]
	AX	Flaxseed	Seed coat epidermal cells	Mature seed	Mucilage [39]

Arabinoxylan (AX); homogalacturonan HG; rhamnogalacturonan I (RGI); galactoglucomannan (GGM). Blank cells correspond to absence of experimental data.
